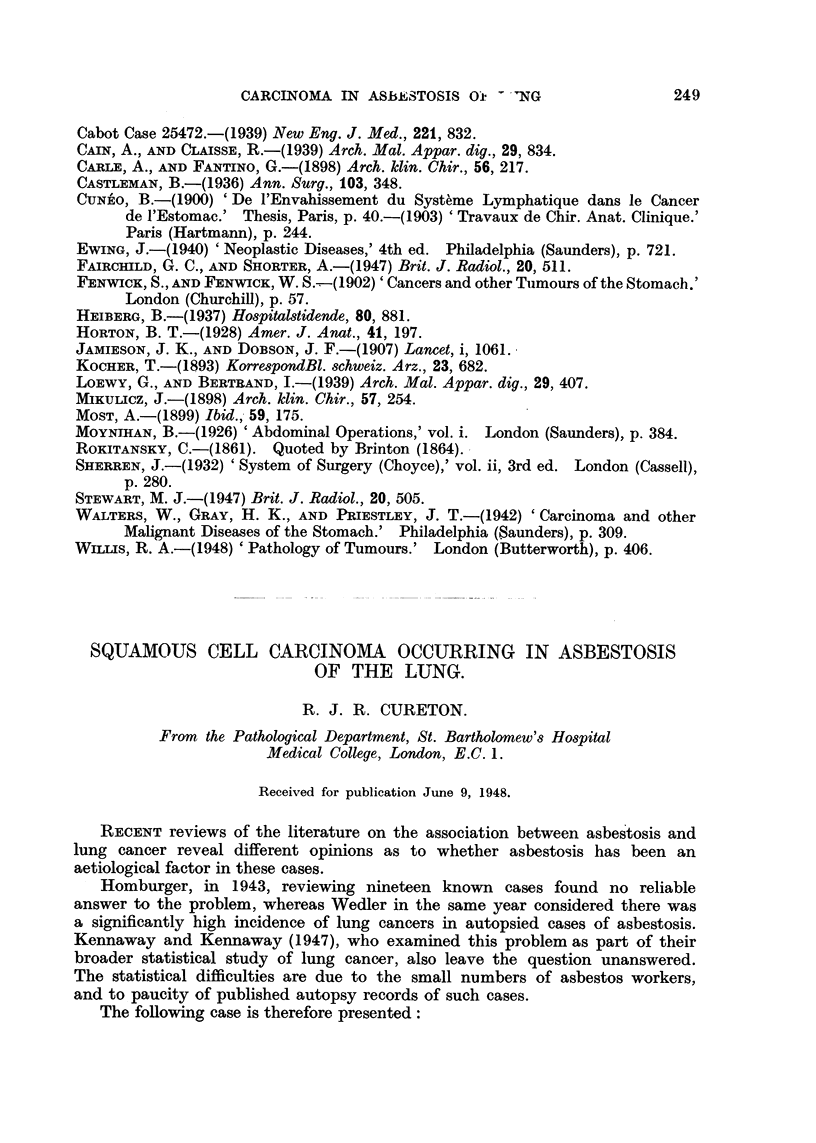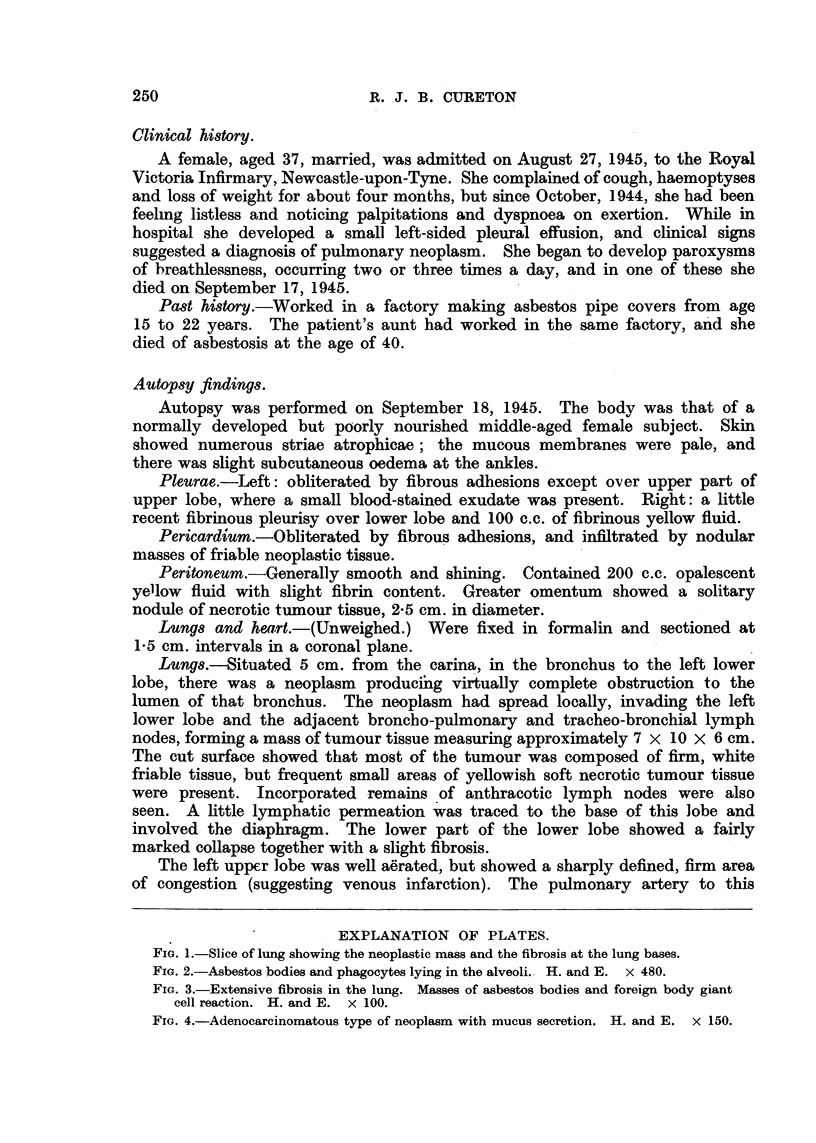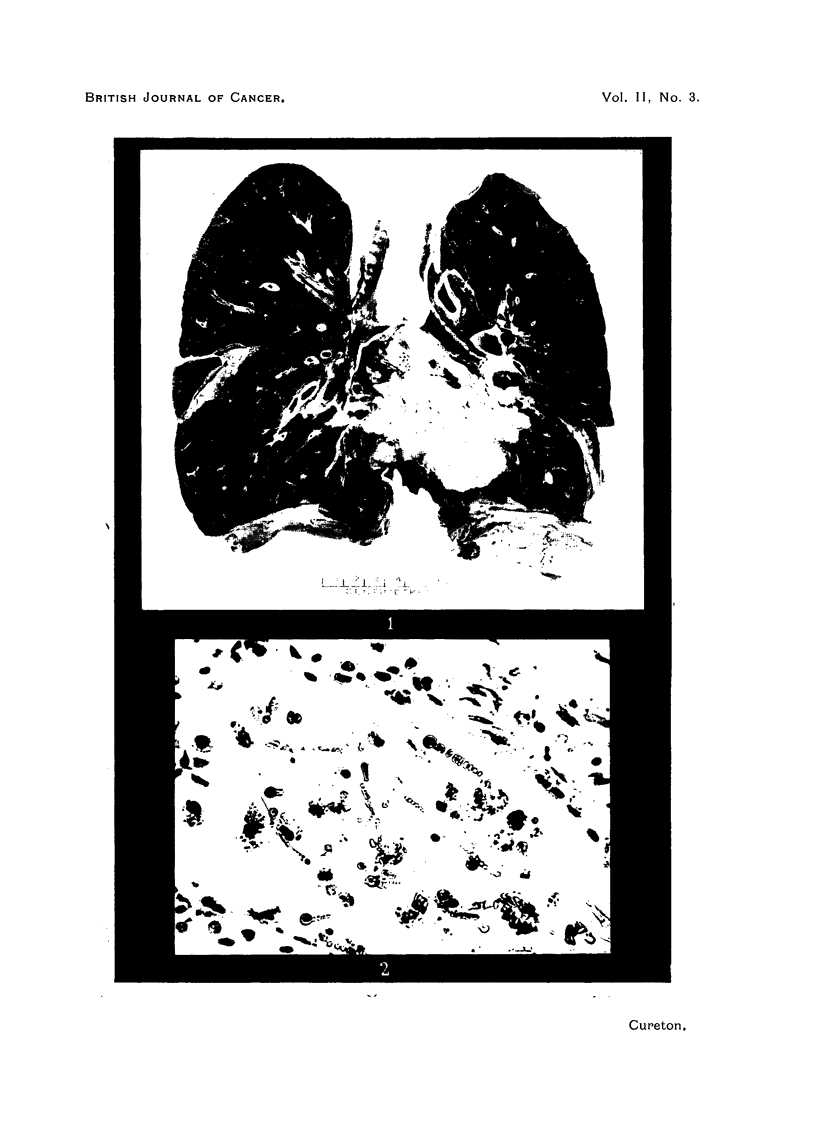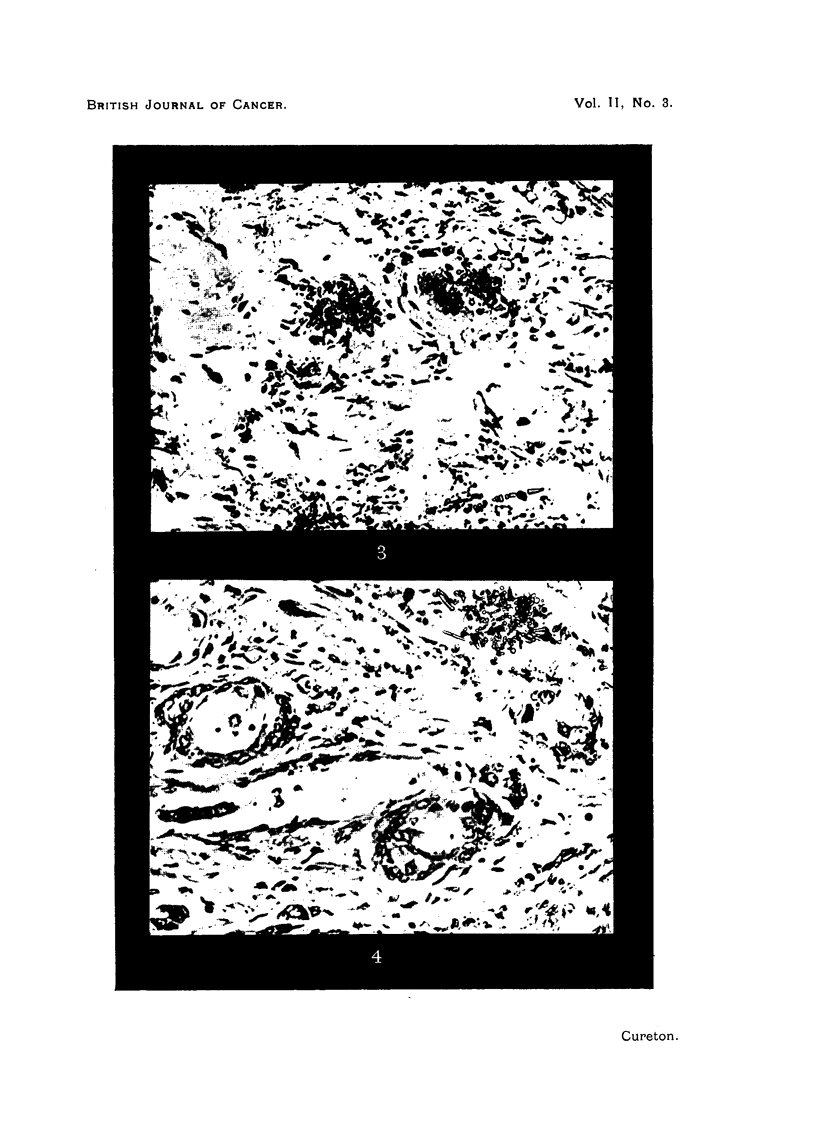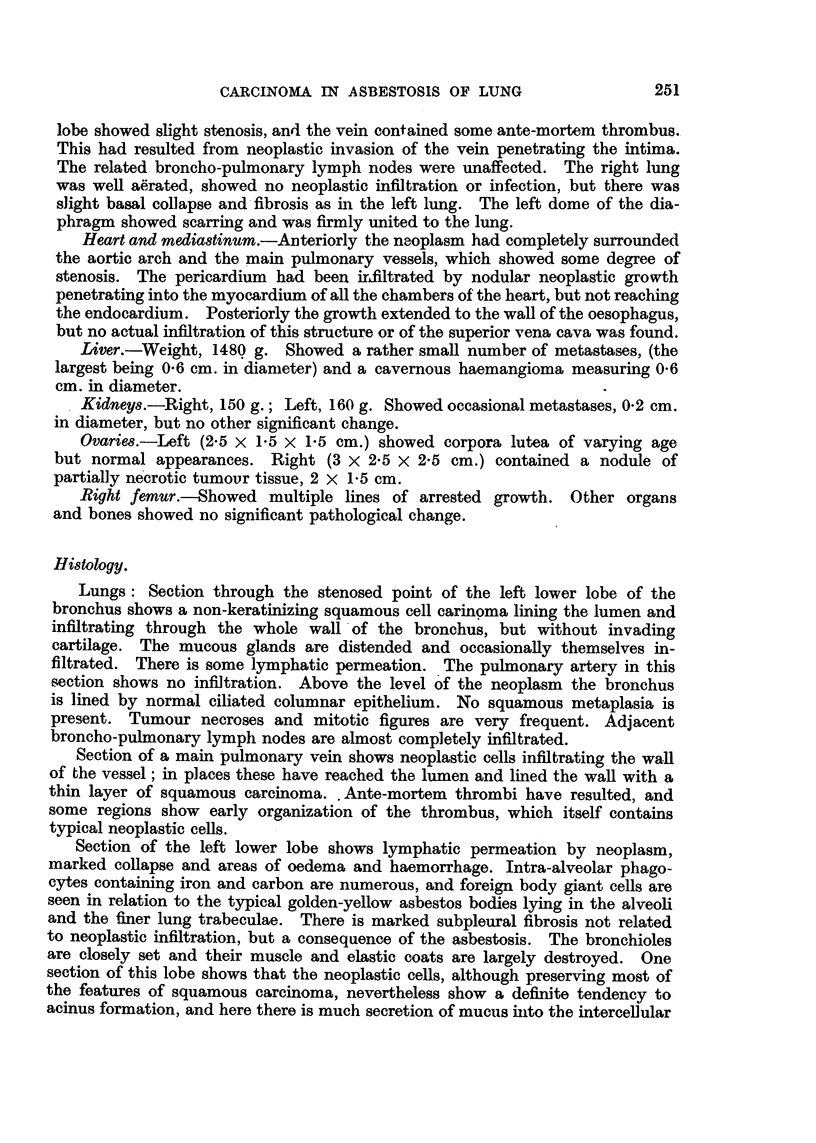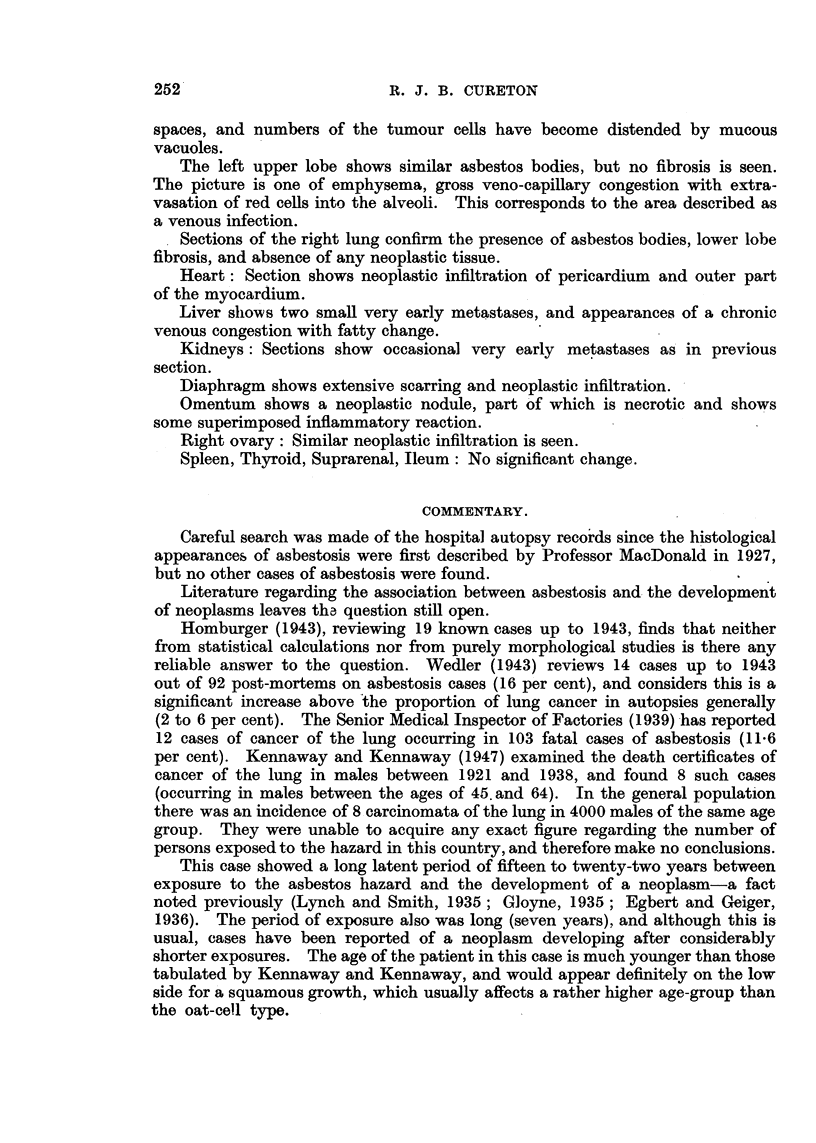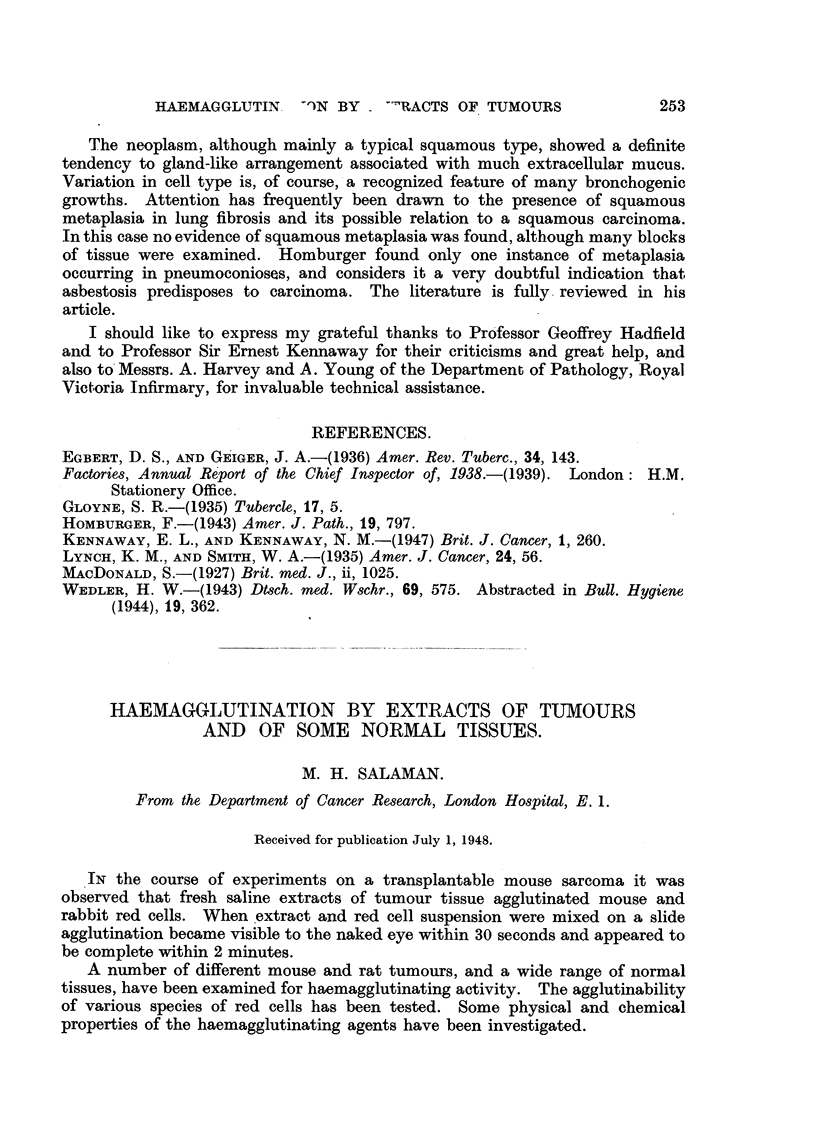# Squamous Cell Carcinoma Occurring in Asbestosis of the Lung

**DOI:** 10.1038/bjc.1948.29

**Published:** 1948-09

**Authors:** R. J. R. Cureton

## Abstract

**Images:**


### SQUAMOUS CELL CARCINOMA OCCURRING IN ASBESTOSIS OF THE LUNG.

R. J. R. CURETON.

*From the Pathological Department, St. Bartholomew's Hospital Medical College, London, E.C. 1.*

Received for publication June 9, 1948.

RECENT reviews of the literature on the association between asbestosis and lung cancer reveal different opinions as to whether asbestosis has been an aetiological factor in these cases.

Homburger, in 1943, reviewing nineteen known cases found no reliable answer to the problem, whereas Wedler in the same year considered there was a significantly high incidence of lung cancers in autopsied cases of asbestosis. Kennaway and Kennaway (1947), who examined this problem as part of their broader statistical study of lung cancer, also leave the question unanswered. The statistical difficulties are due to the small numbers of asbestos workers, and to paucity of published autopsy records of such cases.

The following case is therefore presented :

*Clinical history.*

A female, aged 37, married, was admitted on August 27, 1945, to the Royal Victoria Infirmary, Newcastle-upon-Tyne. She complained of cough, haemoptyses and loss of weight for about four months, but since October, 1944, she had been feeling listless and noticing palpitations and dyspnoea on exertion. While in hospital she developed a small left-sided pleural effusion, and clinical signs suggested a diagnosis of pulmonary neoplasm. She began to develop paroxysms of breathlessness, occurring two or three times a day, and in one of these she died on September 17, 1945.

*Past history.*—Worked in a factory making asbestos pipe covers from age 15 to 22 years. The patient's aunt had worked in the same factory, and she died of asbestosis at the age of 40.

*Autopsy findings.*

Autopsy was performed on September 18, 1945. The body was that of a normally developed but poorly nourished middle-aged female subject. Skin showed numerous striae atrophicae ; the mucous membranes were pale, and there was slight subcutaneous oedema at the ankles.

*Pleurae.*—Left: obliterated by fibrous adhesions except over upper part of upper lobe, where a small blood-stained exudate was present. Right: a little recent fibrinous pleurisy over lower lobe and 100 c.c. of fibrinous yellow fluid.

*Pericardium.*—Obliterated by fibrous adhesions, and infiltrated by nodular masses of friable neoplastic tissue.

*Peritoneum.*—Generally smooth and shining. Contained 200 c.c. opalescent yellow fluid with slight fibrin content. Greater omentum showed a solitary nodule of necrotic tumour tissue, 2·5 cm. in diameter.

*Lungs and heart.*—(Unweighed.) Were fixed in formalin and sectioned at 1·5 cm. intervals in a coronal plane.

*Lungs.*—Situated 5 cm. from the carina, in the bronchus to the left lower lobe, there was a neoplasm producing virtually complete obstruction to the lumen of that bronchus. The neoplasm had spread locally, invading the left lower lobe and the adjacent broncho-pulmonary and tracheo-bronchial lymph nodes, forming a mass of tumour tissue measuring approximately 7 × 10 × 6 cm. The cut surface showed that most of the tumour was composed of firm, white friable tissue, but frequent small areas of yellowish soft necrotic tumour tissue were present. Incorporated remains of anthracotic lymph nodes were also seen. A little lymphatic permeation was traced to the base of this lobe and involved the diaphragm. The lower part of the lower lobe showed a fairly marked collapse together with a slight fibrosis.

The left upper lobe was well aërated, but showed a sharply defined, firm area of congestion (suggesting venous infarction). The pulmonary artery to this

---

EXPLANATION OF PLATES.

FIG. 1.—Slice of lung showing the neoplastic mass and the fibrosis at the lung bases.

FIG. 2.—Asbestos bodies and phagocytes lying in the alveoli. H. and E. × 480.

FIG. 3.—Extensive fibrosis in the lung. Masses of asbestos bodies and foreign body giant cell reaction. H. and E. × 100.

FIG. 4.—Adenocarcinomatous type of neoplasm with mucus secretion. H. and E. × 150.

1

2

3

4

Cureton.

lobe showed slight stenosis, and the vein contained some ante-mortem thrombus. This had resulted from neoplastic invasion of the vein penetrating the intima. The related broncho-pulmonary lymph nodes were unaffected. The right lung was well aërated, showed no neoplastic infiltration or infection, but there was slight basal collapse and fibrosis as in the left lung. The left dome of the diaphragm showed scarring and was firmly united to the lung.

*Heart and mediastinum.*—Anteriorly the neoplasm had completely surrounded the aortic arch and the main pulmonary vessels, which showed some degree of stenosis. The pericardium had been infiltrated by nodular neoplastic growth penetrating into the myocardium of all the chambers of the heart, but not reaching the endocardium. Posteriorly the growth extended to the wall of the oesophagus, but no actual infiltration of this structure or of the superior vena cava was found.

*Liver.*—Weight, 1480 g. Showed a rather small number of metastases, (the largest being 0·6 cm. in diameter) and a cavernous haemangioma measuring 0·6 cm. in diameter.

*Kidneys.*—Right, 150 g.; Left, 160 g. Showed occasional metastases, 0·2 cm. in diameter, but no other significant change.

*Ovaries.*—Left (2·5 × 1·5 × 1·5 cm.) showed corpora lutea of varying age but normal appearances. Right (3 × 2·5 × 2·5 cm.) contained a nodule of partially necrotic tumour tissue, 2 × 1·5 cm.

*Right femur.*—Showed multiple lines of arrested growth. Other organs and bones showed no significant pathological change.

*Histology.*

Lungs: Section through the stenosed point of the left lower lobe of the bronchus shows a non-keratinizing squamous cell carinoma lining the lumen and infiltrating through the whole wall of the bronchus, but without invading cartilage. The mucous glands are distended and occasionally themselves infiltrated. There is some lymphatic permeation. The pulmonary artery in this section shows no infiltration. Above the level of the neoplasm the bronchus is lined by normal ciliated columnar epithelium. No squamous metaplasia is present. Tumour necroses and mitotic figures are very frequent. Adjacent broncho-pulmonary lymph nodes are almost completely infiltrated.

Section of a main pulmonary vein shows neoplastic cells infiltrating the wall of the vessel; in places these have reached the lumen and lined the wall with a thin layer of squamous carcinoma. Ante-mortem thrombi have resulted, and some regions show early organization of the thrombus, which itself contains typical neoplastic cells.

Section of the left lower lobe shows lymphatic permeation by neoplasm, marked collapse and areas of oedema and haemorrhage. Intra-alveolar phagocytes containing iron and carbon are numerous, and foreign body giant cells are seen in relation to the typical golden-yellow asbestos bodies lying in the alveoli and the finer lung trabeculae. There is marked subpleural fibrosis not related to neoplastic infiltration, but a consequence of the asbestosis. The bronchioles are closely set and their muscle and elastic coats are largely destroyed. One section of this lobe shows that the neoplastic cells, although preserving most of the features of squamous carcinoma, nevertheless show a definite tendency to acinus formation, and here there is much secretion of mucus into the intercellular spaces, and numbers of the tumour cells have become distended by mucous vacuoles.

The left upper lobe shows similar asbestos bodies, but no fibrosis is seen. The picture is one of emphysema, gross veno-capillary congestion with extravasation of red cells into the alveoli. This corresponds to the area described as a venous infection.

Sections of the right lung confirm the presence of asbestos bodies, lower lobe fibrosis, and absence of any neoplastic tissue.

Heart: Section shows neoplastic infiltration of pericardium and outer part of the myocardium.

Liver shows two small very early metastases, and appearances of a chronic venous congestion with fatty change.

Kidneys: Sections show occasional very early metastases as in previous section.

Diaphragm shows extensive scarring and neoplastic infiltration.

Omentum shows a neoplastic nodule, part of which is necrotic and shows some superimposed inflammatory reaction.

Right ovary: Similar neoplastic infiltration is seen.

Spleen, Thyroid, Suprarenal, Ileum: No significant change.

### COMMENTARY.

Careful search was made of the hospital autopsy records since the histological appearances of asbestosis were first described by Professor MacDonald in 1927, but no other cases of asbestosis were found.

Literature regarding the association between asbestosis and the development of neoplasms leaves the question still open.

Homburger (1943), reviewing 19 known cases up to 1943, finds that neither from statistical calculations nor from purely morphological studies is there any reliable answer to the question. Wedler (1943) reviews 14 cases up to 1943 out of 92 post-mortems on asbestosis cases (16 per cent), and considers this is a significant increase above the proportion of lung cancer in autopsies generally (2 to 6 per cent). The Senior Medical Inspector of Factories (1939) has reported 12 cases of cancer of the lung occurring in 103 fatal cases of asbestosis (11·6 per cent). Kennaway and Kennaway (1947) examined the death certificates of cancer of the lung in males between 1921 and 1938, and found 8 such cases (occurring in males between the ages of 45 and 64). In the general population there was an incidence of 8 carcinomata of the lung in 4000 males of the same age group. They were unable to acquire any exact figure regarding the number of persons exposed to the hazard in this country, and therefore make no conclusions.

This case showed a long latent period of fifteen to twenty-two years between exposure to the asbestos hazard and the development of a neoplasm—a fact noted previously (Lynch and Smith, 1935; Gloyne, 1935; Egbert and Geiger, 1936). The period of exposure also was long (seven years), and although this is usual, cases have been reported of a neoplasm developing after considerably shorter exposures. The age of the patient in this case is much younger than those tabulated by Kennaway and Kennaway, and would appear definitely on the low side for a squamous growth, which usually affects a rather higher age-group than the oat-cell type.

The neoplasm, although mainly a typical squamous type, showed a definite tendency to gland-like arrangement associated with much extracellular mucus. Variation in cell type is, of course, a recognized feature of many bronchogenic growths. Attention has frequently been drawn to the presence of squamous metaplasia in lung fibrosis and its possible relation to a squamous carcinoma. In this case no evidence of squamous metaplasia was found, although many blocks of tissue were examined. Homburger found only one instance of metaplasia occurring in pneumoconioses, and considers it a very doubtful indication that asbestosis predisposes to carcinoma. The literature is fully reviewed in his article.

I should like to express my grateful thanks to Professor Geoffrey Hadfield and to Professor Sir Ernest Kennaway for their criticisms and great help, and also to Messrs. A. Harvey and A. Young of the Department of Pathology, Royal Victoria Infirmary, for invaluable technical assistance.

### REFERENCES.

Egbert, D. S., and Geiger, J. A.—(1936) *Amer. Rev. Tuberc.*, **34**, 143.
*Factories, Annual Report of the Chief Inspector of, 1938.*—(1939). London: H.M. Stationery Office.
Gloyne, S. R.—(1935) *Tubercle*, **17**, 5.
Homburger, F.—(1943) *Amer. J. Path.*, **19**, 797.
Kennaway, E. L., and Kennaway, N. M.—(1947) *Brit. J. Cancer*, **1**, 260.
Lynch, K. M., and Smith, W. A.—(1935) *Amer. J. Cancer*, **24**, 56.
MacDonald, S.—(1927) *Brit. med. J.*, ii, 1025.
Wedler, H. W.—(1943) *Dtsch. med. Wschr.*, **69**, 575. Abstracted in *Bull. Hygiene* (1944), **19**, 362.